# Biodegradable Microrobots and Their Biomedical Applications: A Review

**DOI:** 10.3390/nano13101590

**Published:** 2023-05-09

**Authors:** Jinxin Li, Jiangfan Yu

**Affiliations:** 1 School of Science and Engineering, The Chinese University of Hong Kong, Shenzhen 518172, China; jinxinli@link.cuhk.edu.cn; 2 Shenzhen Institute of Artificial Intelligence and Robotics for Society, Shenzhen 518172, China

**Keywords:** biodegradable, micro/nanorobots, micro/nanomotors, biomedicine, targeted delivery

## Abstract

During recent years, microrobots have drawn extensive attention owing to their good controllability and great potential in biomedicine. Powered by external physical fields or chemical reactions, these untethered microdevices are promising candidates for in vivo complex tasks, such as targeted delivery, imaging and sensing, tissue engineering, hyperthermia, and assisted fertilization, among others. However, in clinical use, the biodegradability of microrobots is significant for avoiding toxic residue in the human body. The selection of biodegradable materials and the corresponding in vivo environment needed for degradation are increasingly receiving attention in this regard. This review aims at analyzing different types of biodegradable microrobots by critically discussing their advantages and limitations. The chemical degradation mechanisms behind biodegradable microrobots and their typical applications are also thoroughly investigated. Furthermore, we examine their feasibility and deal with the in vivo suitability of different biodegradable microrobots in terms of their degradation mechanisms; pathological environments; and corresponding biomedical applications, especially targeted delivery. Ultimately, we highlight the prevailing obstacles and perspective solutions, ranging from their manufacturing methods, control of movement, and degradation rate to insufficient and limited in vivo tests, that could be of benefit to forthcoming clinical applications.

## 1. Introduction

**Figure 1:**
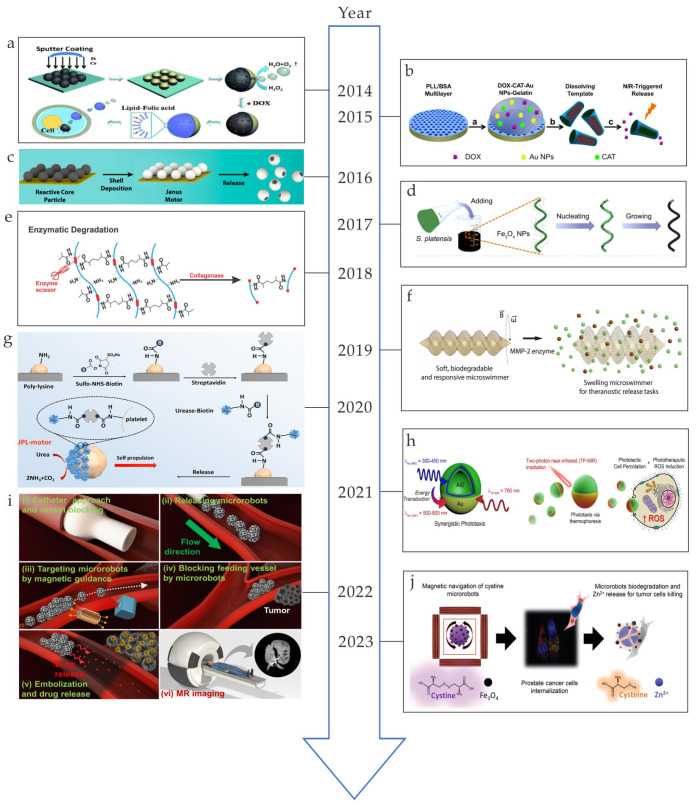
(**a**) Preparation of Janus mesoporous silica nanoparticle microrobots (reprinted with permission from Ref. [25]). (**b**) Fabrication and near-infrared (NIR)-triggered release process of microrobots (reprinted with permission from Ref. [26]). (**c**) Fabrication of Janus microrobots (reprinted with permission from Ref. [27]). (**d**) Illustration of the immersion coating technique applied to Spirulina platensis using an Fe_3_O_4_ nanoparticle suspension (reprinted with permission from Ref. [28]). (**e**) Enzymatic degradation process of GelMA (reprinted with permission from Ref. [29]). (**f**) Illustration of enzymatic breakdown of microswimmers (reprinted with permission from Ref. [30]). (**g**) Diagram of the preparation process of Janus platelet microrobots (reprinted with permission from Ref. [31]). (**h**) Development of phototherapeutic microrobots utilizing AIE–based synergistic design (reprinted with permission from Ref. [32]). (i) Illustration of the steps of liver cancer therapy. The white dotted arrow in (iii) indicates the motion path of the microrobots and the yellow dotted arrows in (iii) indicate the magnetic guidance (reprinted with permission from Ref. [33]). (**j**) Schematic illustration of the delivering process of microrobots and the release of Zn^2+^ (reprinted with permission from Ref. [34]).

In the past decade, microrobots have been widely investigated in different areas [1,2]. Different from traditional robots, these tiny robots have simple structures, e.g., helical and sphere shapes, and, due to their small size, they are powered by external physical fields [1]. Different kinds of materials and power types have been applied, such as magnetic fields [3,4,5], optical fields [6], and electrical fields [7]. Although large amounts of microrobots are developed for biomedical applications [8,9,10,11], the issue of biodegradation remains to be investigated [12,13,14,15,16,17,18,19]. After microrobots complete their in vivo tasks, e.g., targeted drug delivery, thrombolysis, and hyperthermia, they are left in the body, which could be cytotoxic and may induce unexpected harmful results [20,21]. In this case, microrobots made of biodegradable materials have become the trend in healthcare and biomedical applications [22,23,24], which has boosted a large amount of research in the past few years. The milestones in this research field are shown in Figure 1. In 2014, Janus mesoporous silica microrobots that could be propelled by oxygen bubbles generated by decomposing hydrogen were developed (Figure 1a) [25]. In 2015, biodegradable protein-based microrobots that could effectively kill cancer cells through the targeted delivery of doxorubicin were presented (Figure 1b) [26]. In 2016, transient self-destroyed micromotors that could disappear completely in a biological environment were fabricated (Figure 1c) [27]. The different corrosion rates of the core–shell components of Janus microrobots result in their degradation. In 2017, biodegradable magnetic microswimmers that had specific cytotoxicity to cancer cells were reported (Figure 1d) [28]. In 2018, noncytotoxic biodegradable soft helical swimmers that could be degraded completely by collagenase were developed (Figure 1e) [29]. In 2019, biodegradable hydrogel-based microswimmers that could be degraded within 118 h and that delivered therapeutic cargo to the target area were fabricated (Figure 1f) [30]. In 2020, cell-based biodegradable microrobots that could be powered by urea fuel and that targeted cancer cells were developed (Figure 1g) [31]. In 2021, biodegradable photoactivated nanomotors decorated with aggregation-induced emission (AIE) motifs that could be used for enhanced phototherapy were fabricated (Figure 1h) [32]. In 2022, real-time trackable polylactic-co-glycolic acid (PLGA)- and gelatin-based microrobots that could degrade slowly after targeted vessel embolization were presented (Figure 1i) [33]. In 2023, biodegradable self-assembly magnetically driven cystine microrobots that could perform a targeted delivery of Zn^2+^ to kill prostate tumor cells were reported (Figure 1j) [34].

The biodegradability of microrobots still requires extensive investigation. Different types of biodegradable materials have been analyzed and discussed [35], lacking a further summary of the degradation mechanism. Biodegradability is closely related to drug release and targeted delivery and also requires a timely review. In this review, the progress in the field of microrobots consisting of biodegradable materials is summarized, and, meanwhile, the degradation mechanism of microrobots and their corresponding biomedical applications are introduced [36,37,38,39,40].

## 2. Biodegradable Materials

The materials used to construct microrobots play a critical role in their functionality and overall performance [41,42,43,44,45]. One of the key challenges in microrobot design is selecting materials that can be small enough to fit within the desired size constraints, possess the necessary mechanical and magnetic properties to carry out their intended tasks, and can be degraded naturally in the human body.

### 2.1. Metal-Based Microrobots

**Figure 2:**
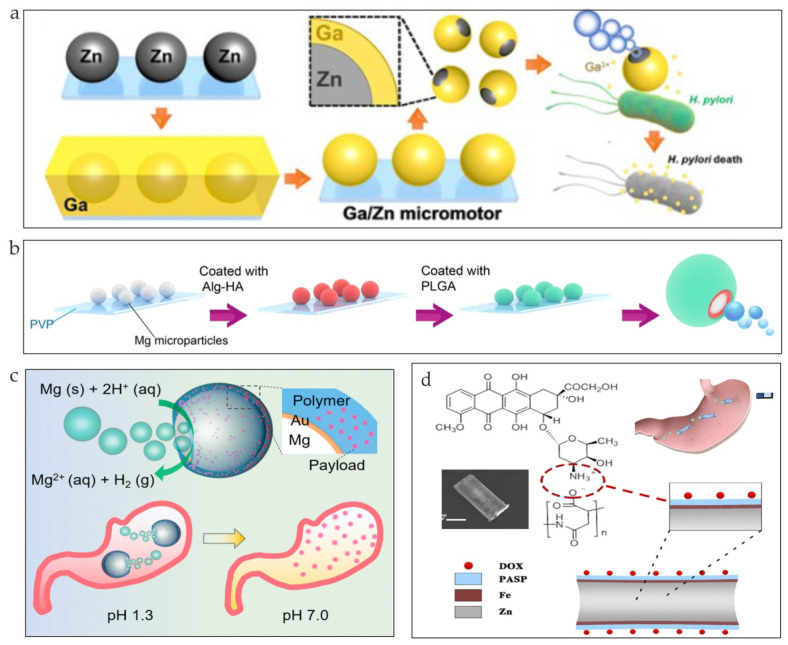
(**a**) Illustration of the fabrication and antibacterial mechanism of Janus Ga/Zn microrobots (reprinted with permission from Ref. [46]). (**b**) Characterization and fabrication process of Mg–HA microrobots (reprinted with permission from Ref. [47]). (**c**) Illustrations of magnesium-based microrobots powered by acid and their method for neutralizing acid (reprinted with permission from Ref. [48]). (**d**) Illustrative diagram of a DOX/PASP/Fe–Zn microrobot and its application for effective localization in the stomach (reprinted with permission from Ref. [49]).

Reactive metals, such as Mg, Zn, and Ga, have good biodegradability and biocompatibility and can react with common components, such as water, in the human body. The product of these chemical reactions can act as a force of propulsion by breaking the static equilibrium near the microrobots. As is shown in Figure 2a, Ga/Zn microrobots that were propelled by hydrogen bubbles to treat bacterial infections were fabricated [46]. Hydrogen bubbles were generated through the zinc–acid reaction when the microrobots entered the gastric acid, where pH = 1.5. Mg-based microrobots for rheumatoid arthritis therapy were developed [47]. Mg microparticles were first coated with alginate–HA hydrogel layers and were then coated with biodegradable PLGA layers for stability (Figure 2b). Other Mg-based microrobots coated with gold layers and drug-encapsulated pH-sensitive polymer layers were fabricated (Figure 2c) [48]. These microrobots can actively neutralize the acidic environment and break into biocompatible components that are safe for the body. Microrobots that consist of poly(aspartic acid) (PASP) microtubes, Fe layers, and a core of Zn can display self-propelling movement by converting the chemical energy produced by the Zn–acid reaction as shown in Figure 2d [49].

### 2.2. Polymer-Based Microrobots

**Figure 3:**
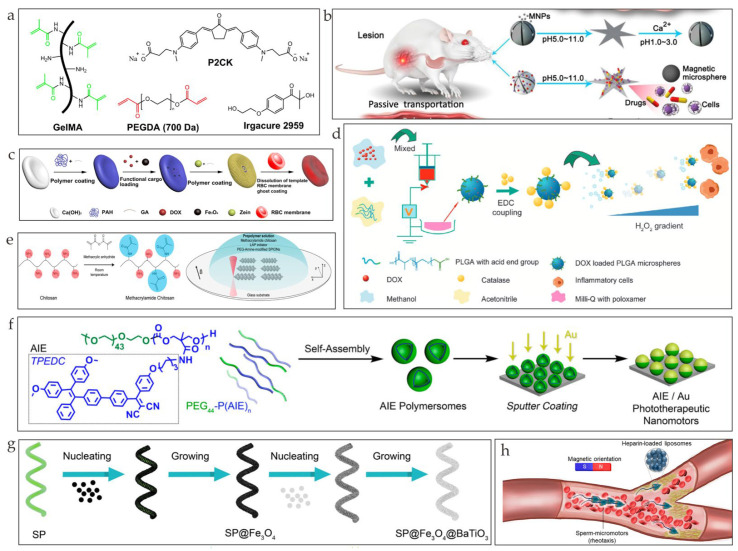
(**a**) Chemicals employed in this study (reprinted with permission from Ref. [29]). (**b**) Schematic illustration of ionic shape-morphing microrobotic end effectors (ISME) in the digestive system (reprinted with permission from Ref. [61]). (**c**) Schematic illustration of the fabrication process of an RBCM (reprinted with permission from Ref. [60]). (**d**) Diagrammatic representation of the preparation of motored PLGA particles (reprinted with permission from Ref. [62]). (**e**) Fabrication of photo-cross-linkable methacrylamide chitosan from natural chitosan (reprinted with permission from Ref. [63]). (**f**) Hybrid AIE/Au microrobots using biodegradable copolymers with AIE−genic compound comprising both tetraphenylethylene and dicyanovinyl moieties (reprinted with permission from Ref. [32]). (**g**) Schematic illustration of fabricating microrobots based on Spirulina platensis via dip coating in a suspension of Fe_3_O_4_ and BaTiO_3_ nanoparticles (reprinted with permission from Ref. [64]). (**h**) Schematic illustration of sperm microrobots swimming against flowing blood and delivering heparin cargo (reprinted with permission from Ref. [65]).

The properties of different kinds of polymers, such as biopolymers produced by microorganisms, hydrogel, alginate, and other artificial polymers, have long been investigated [50,51,52,53]. Different fabrication methods of microrobots can be applied, including two-photon polymerization (2PP) 3D printing [54,55,56,57,58] and bioprinting [59]. Helical microswimmers printed through the 2PP of gelatin methacryloyl (GelMA) are empowered to be magnetic by incubating Fe_3_O_4_ nanoparticles (Figure 3a) [29]. Microrobots with an alginate hydrogel microstructure fabricated through the microelectrode-based method were presented (Figure 3b). As is shown in Figure 3c, magnetic red-blood-cell-mimetic microrobots (RBCMs) that had frameworks of the natural protein zein loaded with doxorubicin (DOX) and Fe_3_O_4_ nanoparticles and that were fused with an isolated red blood cell membrane were constructed [60,61]. DOX-loaded grafted PLGA microrobots with enzyme coverage were fabricated (Figure 3d) [62]. Chitosan-based helical microswimmers that were fabricated through two-photon-based 3D printing and that were loaded with a certain amount of magnetic nanoparticles were proposed (Figure 3e) [63]. Block-copolymer-based phototactic microrobots that were decorated with AIE motifs for enhanced phototherapy were reported (Figure 3f) [32].

### 2.3. Biohybrid Microrobots

Biohybrid microrobots consist of biological cells and synthetic materials [66]. This kind of microrobot can be actuated both through biological means and artificial means [11]. *Spirulina* is frequently taken into consideration when constructing these cell-based microrobots owing to its property of tailored biodegradation [67]. For example, microrobots that utilize *Spirulina platensis* (*S. platensis*) as their framework and incorporate Fe_3_O_4_ nanoparticles and BaTiO_3_ nanoparticles as functional components can be propelled by a magnetic rotational field (Figure 3g) [64]. Another multicellular magnetically driven microrobot engineered from *Spirulina* can achieve the loading of molecular cargos through the dehydration and rehydration of *Spirulina* cells [68]. Because of the high propulsion force provided by their flagellum, sperm cells are also recognized as promising candidates for biohybrid microrobots. Nonmotile sperm cells and magnetic nanoparticles were combined through electrostatic self-assembly to formulate biocompatible sperm-templated soft magnetic microrobots [69]. Furthermore, once magnetized, individual sperm microrobots can form train-like carriers to perform targeted delivery, which has the potential to treat blood clots (Figure 3h) [65]. Other cell-based biodegradable microrobots, such as human-adipose-derived mesenchymal-stem-cell-based microrobots [70] and neutrophil-based microrobots [71], inherit the biological characteristics of cells to enhance their targeting efficacy and to reduce the inflammation response.

## 3. Degradation Mechanism

Biodegradable microrobots are designed to degrade over time in the body, allowing them to be used for targeted drug delivery and other medical applications without leaving behind any harmful residues. The degradation mechanism of biodegradable microrobots depends on several factors, including the materials, the size and shape of the microrobots, and the environment in which they are deployed. Degradation mechanisms include enzymatic degradation, oxidation or hydrolysis under acidic or alkaline conditions, and photodegradation. This article mainly focuses on pH degradation and enzymatic degradation.

### 3.1. pH Degradation

**Figure 4:**
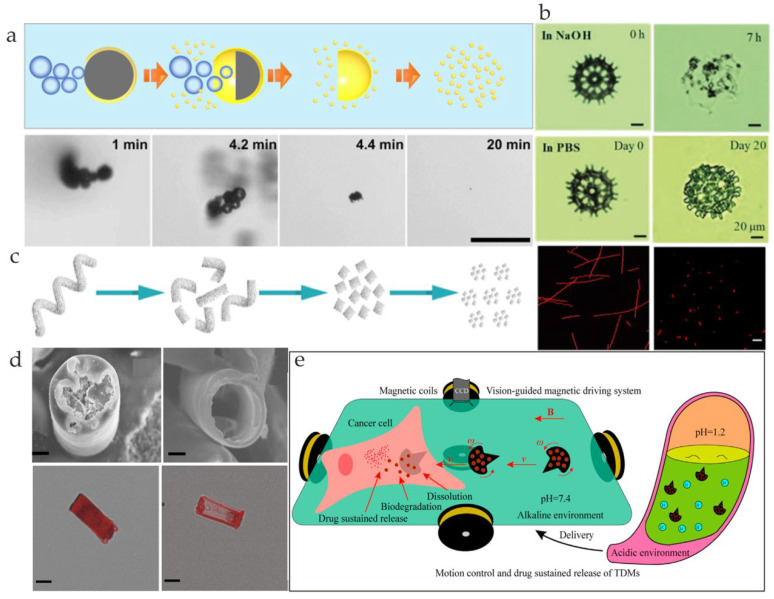
(**a**) Diagram demonstrating the breakdown mechanism of a Ga/Zn microrobot in gastric acid (reprinted with permission from Ref. [46]). (**b**) Degradation process of microrobots in NaOH and PBS solution (reprinted with permission from Ref. [73]). (**c**) A graphical representation of the degradation process of the SP@Fe_3_O_4_@BaTiO_3_ micromotor (reprinted with permission from Ref. [64]). (**d**) Scanning electron microscopy images and confocal fluorescence images of DOX/PASP/Fe–Zn microrobots before and after the metal–acid reaction (reprinted with permission from Ref. [49]). (**e**) Schematic of the fabrication process, motion control, and sustained release of drugs by TDMs (reprinted with permission from Ref. [74]).

There are two kinds of mechanisms for pH degradation. One is degradation in an acidic environment, and the other one is degradation under an alkaline condition. Most metal-based microrobots are degraded through a metal–acid reaction. The basic principle is M (s) + H^+^ (aq) → M^+^ (aq) + H_2_ (g) (M is the reactive metal). As shown in Figure 4a, the Janus Ga/Zn microrobots were immersed in gastroenteric acid (pH = 1.5), and the speed of the degradation process was examined [46]. The Zn core was degraded completely after movement, and the remaining Ga shell was degraded within 15 min. The release rate of the Zn and Ga cations was equal to their degradation rate. A DOX/PASP/Fe–Zn microrobot can be completely consumed in the acidic stomach environment and digestive tract (Figure 4d) [49]. Moreover, the metabolites are biocompatible trace elements that are harmless to the human body. It has been pointed out that the potential difference between Zn and Fe is lower than that of the traditional Au-Zn. Therefore, these microrobots have a longer navigation lifetime. A chemotaxis-driven 2D nanosheet that collapsed and disintegrated at a higher efficiency under lysosomal acidity (pH = 5) than in a neutral solution (pH = 7) was discovered [72]. However, the specific disintegration process, such as the toxicity of the metabolites and the speed of degradation, was not discussed. Through controlled experiments, some biodegradable microrobots proved that they had better degradation performances in alkaline environments than PBS environments [73]. Furthermore, in vivo tests were carried out to examine the degradability of microrobots in the subcutaneous tissues of nude mice, and it was found that they can also be successfully degraded (Figure 4b). Spirulina platensis (SP)@Fe_3_O_4_@BaTiO_3_ microrobots can break down into small pieces after 192 h of incubation in Dulbecco’s phosphate-buffered saline (DPBS) solution (Figure 4c) [64]. As shown in Figure 4e, double-layer drug-loaded microrobots (TDMs) can dissolve gradually in an alkaline environment (pH = 7.4), and the degradation product has little cytotoxicity [74].

### 3.2. Protease

**Figure 5:**
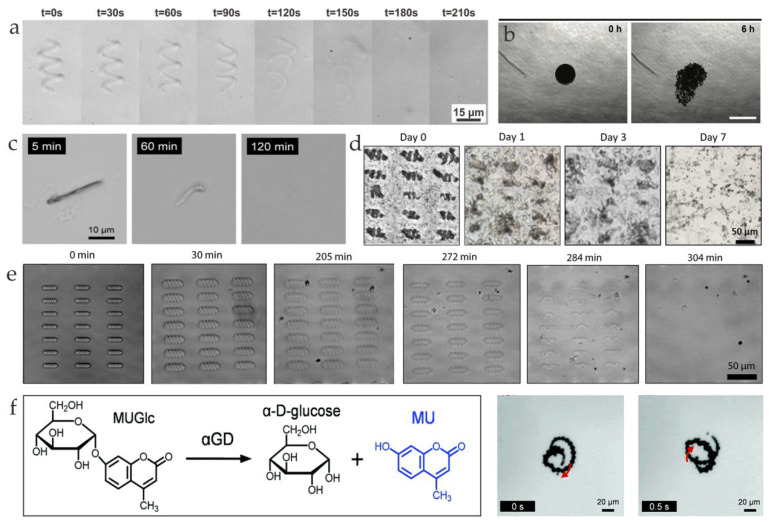
(**a**) Degradation of a GelMA helical microstructure in a collagenase solution (0.1 mg mL^−1^) (reprinted with permission from Ref. [29]). (**b**) Enzymatic biodegradation and magnetic retrieval of SPIONs from the GelMA microrobot in the absence of hNTSCs (reprinted with permission from Ref. [75]). (**c**) Transformation of the morphology of Avi/bUre microtube in pronase solution at 37 °C. (reprinted with permission from Ref. [77]). (**d**) Optical images displaying the degradation process of magnetoelectric (ME) soft helical microswimmers after being cultured with cells for 0, 1, 3, and 7 days (reprinted with permission from Ref. [76]). (**e**) Differential interference contrast (DIC) images of a degrading microswimmer array with 4 μg/mL of enzyme (reprinted with permission from Ref. [30]) (**f**) Microscopic observations of self-propulsion of aGD/Cat MTs through the jetting of O_2_ bubbles in phosphate buffer (PB) solution and hydrolysis reaction of MTs. The red arrows indicate the direction of O_2_ bubbles (reprinted with permission from Ref. [78]).

Degradable microrobots with proteases are also common. There are different kinds of proteases, and many polymer-based microrobots can be degraded by proteases secreted by cells. For example, Wang et al. proved that the GelMA microstructure can be efficiently degraded by the enzyme collagenase type II, which is secreted by cells (Figure 5a) [29]. The same group further studied the advantage of a helical shape over a cuboid shape and the attached cell viability of microrobots, confirming their biocompatibility. It was demonstrated that cell-loaded microrobots can be effectively degraded with 0.1 mg mL^−1^ of collagenase type II with a negligible impact on cell viability (Figure 5b) [75]. GelMA integrated with multiferroic nanoparticle microswimmers were also fabricated, which could be gradually degraded by the proteases secreted by surrounding cells [76]. They took seven days to disappear completely (Figure 5d). Moreover, microrobots consisting only of proteins and polypeptides that could be decomposed by proteases were also fabricated [77]. Through incubation in acidic water first and then putting them into the aqueous solution of a protease mixture, the microrobots disappeared completely (Figure 5c). The degradation effect of different concentrations of matrix metalloproteinase was studied, respectively, since this protease was reportedly present at various concentrations in a healthy individual (Figure 5e) [30]. Catalase-driven protein microrobots that could be digested by proteases were developed [78]. It was shown that the four Cat microtubes (MTs) became shorter and more slender in a cocktail of several proteases within two hours (Figure 5f). The complete degradation of milligrippers by the metalloproteinase-2 enzyme took 192 h in the experimental environment [79]. However, a longer duration was expected in the physiological environment because of the lower concentration of enzymes.

### 3.3. Lipase

**Figure 6:**
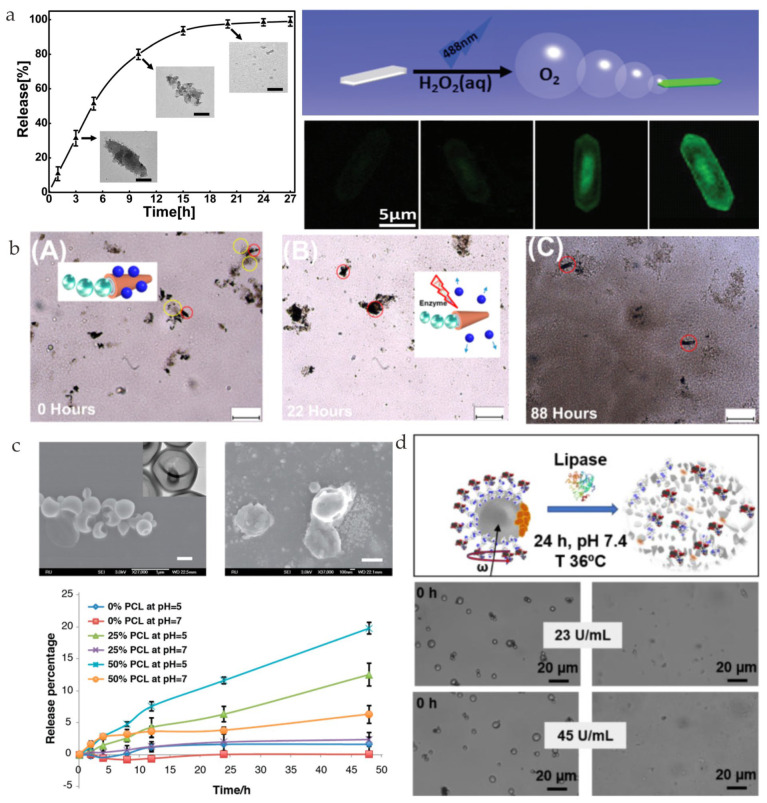
(**a**) The degradation process of catalase and FITC−decorated PCL−SH single crystal (CAT−PCL−FITC) over time and its autonomous movement in H_2_O_2_ solution (reprinted with permission from Ref. [80]). (**b**) The degradation process of PCL−SH nanospheres and PCL−SH/Pt microrobots. The red circles indicate the microrobots and the yellow circles indicate the nanospheres (reprinted with permission from Ref. [81]). (**c**) The formation of pores in stomatocytes before and after degradation (reprinted with permission from Ref. [82]). (**d**) Scheme of the enzyme degradation of PCL−Fe_3_O_4_/PEI@DOX magnetic microrobots after 24 h of treatment with lipase (up) and microscopy images before and after 24 h of enzymatic treatment using lipase concentrations of 23 U/mL and 45 U/mL (down) (reprinted with permission from Ref. [83]).

In addition to proteases, lipases are also widely used as a means of biodegradation. Catalase and an FITC-decorated thiol-terminated polycaprolactone (PCL-SH) single crystal can be almost fully degraded by phosphate-buffered saline (PBS) solution containing lipase after 20 h of incubation (Figure 6a) [80]. PCL-SH microrobots that were subjected to incubation with lipase for six days were developed (Figure 6b) [81]. It is worth noting that the nanospheres loaded by microrobots can also be digested by lipase. The hybrid stomatocytes can easily be degraded by either an acid or lipase (Figure 6c) [82]. PCL-Fe3O4/PEI@DOX magnetic microrobots can be disassembled into small pieces of crystals in the presence of lipase as shown in Figure 6d [83]. With 45 U/mL of porcine pancreas lipase, the microrobots can be disintegrated completely.

The previous literature has shown microrobots made of biodegradable materials that can be degraded by enzymes; however, the details of degradation are still under investigation [84,85]. Other methods of degradation, such as thermal degradation, photodegradation, and degradation through a combination of pH and enzymes, are also being explored by researchers [86,87,88,89]. Overall, by understanding these mechanisms, researchers can design microrobots that degrade at a controlled rate, allowing them to be used for a wide range of biomedical applications.

## 4. Biomedical Application

Microrobots have potential in biomedicine, including potential in microsurgery [1] and enhanced imaging [24,90]. Targeted delivery has emerged as a fast-developing modality of microsurgery in the past decade [91]. Microrobots can be designed to carry payloads to specific locations within the body, such as to tumors, where they can release the payloads. This approach could potentially reduce the amount of medication needed and could minimize side effects since the payloads would only be delivered to the affected area.

### 4.1. Drug Delivery

**Figure 7:**
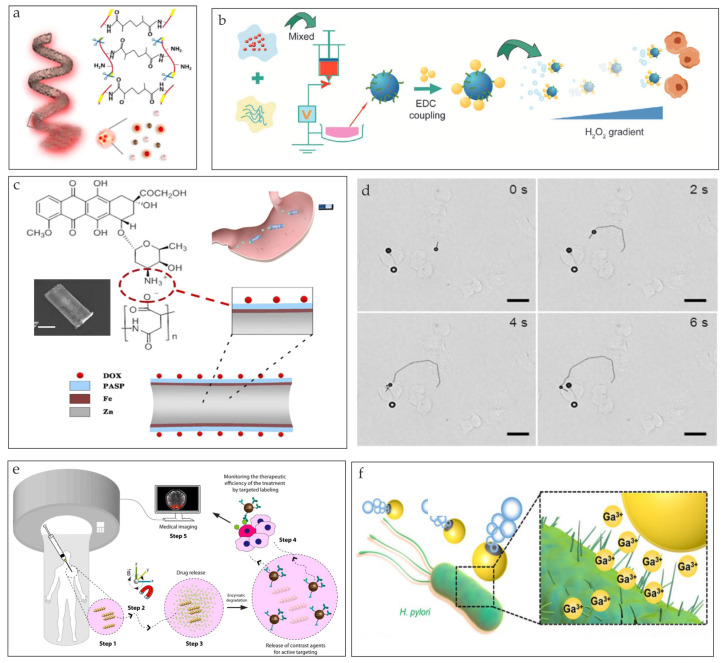
(**a**) Depiction of the degradation process of metal–organic-framework-based microrobots (MOFBOTs) (reprinted with permission from Ref. [97]). (**b**) Preparation of motored PLGA particles (reprinted with permission from Ref. [62]). (**c**) Schematic illustration of drug delivery of DOX/PASP/Fe−Zn microrobots in the stomach (reprinted with permission from Ref. [49]). (**d**) Time-lapse image showing the target location of a microrobot in HeLa cells in 3% H_2_O_2_ at 22 °C (reprinted with permission from Ref. [98]). (**e**) The potential therapeutic use of 3D-printed biodegradable microrobotic swimmers (reprinted with permission from Ref. [30]). (**f**) Graphical representation of the antibacterial activity of Ga/Zn micromotors against *H. pylori* bacteria (reprinted with permission from Ref. [46]).

The whole process of drug delivery can be described as three parts: drug loading, targeted delivery, and drug release. In vitro and in vivo experiments have been done to mitigate the process. For in vitro experiments, investigations have been conducted on the fabrication and actuation methods [92,93,94,95,96]. However, since the requirements of drug delivery, such as the lasting time of drug release, differ in different physiological environments, in vivo tests are necessary. For drug delivery, drug release has a strong connection with the degradation process. This is because payloads are often loaded inside microrobots, and the drug is released when the structural degradation of the microrobots occurs. Fe@ZIF-8 can keep the therapeutic cargo at a physiological pH and can release it only in pathological acidic microenvironments (Figure 7a) [97]. The PLGA microrobots showed reduced burst release and long-term drug delivery behavior, which were good for a periodontal application (Figure 7b) [62]. PASP microrobots can slowly release concentrated DOX payloads onto the stomach wall (Figure 7c) [49]. Since the microrobots can be magnetically located, placing a strong magnet near a particular position on the stomach can achieve targeted drug delivery. An ultrasonic treatment was applied to perform the triggered release of the encapsulated DOX drug in the microrobots (Figure 7d) [98]. Swelling-controlled drug delivery was developed. This method uses the rapid swelling of the microswimmer as a switch for accelerated drug release (Figure 7e) [30]. For the Janus Ga/Zn microrobots, most Zn became Zn^2+^ within 5 min of immersion in gastroenteric acid, and nearly all the Ga became cations in 20 min (Figure 7f) [46]. The released Ga^3+^ served as a drug to kill bacteria. Wu et al. developed microcapsules that contained drug-loaded microrobots [99]. Near-infrared light irradiation can trigger the release of the microrobots. At the same time, the anticancer drug encapsulated in the microrobots can be released.

### 4.2. Cell Delivery

**Figure 8:**
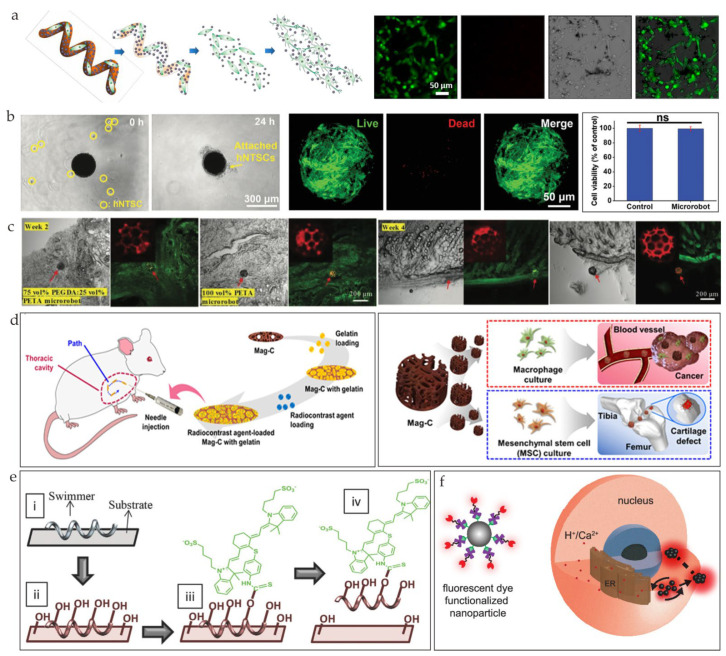
(**a**) Illustration of microswimmer’s degradation process and induced neuronal differentiation of SH-SY5Y cells (reprinted with permission from Ref. [76]). (**b**) Live/dead cell imaging of the hNTSCs on the microrobot (left), images after incubating cells with microswimmers (middle), and evaluation of cell viability (right) (reprinted with permission from Ref. [75]). (**c**) Confocal scans of histological sections of skin tissues implanted with degradable 75 vol% polyethylene glycol diacrylate (PEGDA):25 vol% pentaerythritol triacrylate (PETA) microrobot and hard-to-degrade 100 vol% PETA microrobot. The red arrows indicate the location of the microrbot (reprinted with permission from Ref. [73]). (**d**) Preparation and in vivo locomotion of the radiocontrast-agent-loaded magnetic chitosan microscaffold (Mag-C) for real-time X-ray imaging (left) and schematics of Mag-C containing macrophages and human adipose-derived mesenchymal stem cells (hADMSCs) used for cancer therapy and cartilage regeneration (right) (reprinted with permission from Ref. [101]). (**e**) Conjugation of NIR−797 dyes to ABFs for functionalization (reprinted with permission from Ref. [102]). (**f**) Schematic illustration of fluorescent-dye-coated magnetic nanoparticles and the generation and navigation of swarm inside cell. The black arrows indicate the direction of the magnetic field (reprinted with permission from Ref. [103]).

In an effective and efficient cell delivery process, two key factors are considered, i.e., cell viability and cell adhesion. Targeted cell delivery tends to utilize stem cells that have the ability to proliferate and differentiate. Helical microswimmers can release loaded therapeutic cells and magnetoelectric nanoparticles by degrading themselves when the cells have been delivered to the targeted area (Figure 8a) [76]. Cell attachment without ultraviolet exposure was achieved to avoid cell damage (Figure 8b) [75]. Cell attachment and detachment can also be achieved through temperature variation [100]. During the degradation of cell-loaded microrobots, human nasal turbinate stem cells (hNTSCs) and superparamagnetic iron oxide nanoparticles (SPIONs) are released. To stimulate the differentiation of the neuronal cells, at the same time, an alternating magnetic field is applied to the system. The effect of cell therapy is mainly defined by cell release [73]. Cell release has a direct connection with the degradation of cell-loaded microrobots. Because of the different properties of different cell proteins, cells can both adhere to the microrobots and migrate away from them (Figure 8c). Cells can also be used as drug-loaded biocarriers owing to their ability to target cancer. Macrophages and mesenchymal stem cells were selected for their abilities to target cancer and tissue regeneration (Figure 8d) [101]. The magnetic chitosan microrobots were a suitable environment for cell growth, and, after incubation for five days, the cells adhered harmoniously to the magnetic chitosan microrobots.

### 4.3. Bioimaging

In addition to targeted delivery, microrobots can also be used for imaging enhancement [104,105,106], which could contribute to early-stage diagnostics and could lead to better treatment outcomes. Fluorescence imaging (FI) is a commonly used biomedical imaging tool. Owing to the intrinsic property of autofluorescence and biocompatibility, a Spirulina plant is an ideal material for bioimaging. Microrobots based on *Spirulina Plantensis* were fabricated through dip coating in an Fe_3_O_4_ nanoparticle suspension [28]. These swarms of microrobots can achieve innate fluorescence and can be tracked instantly. By creating a swarm of artificial bacteria flagella (ABFs) labeled with an isothiocyanate dye (NIR-797) (Figure 8e), the Nelson team was able to track the movement of ABFs in the peritoneal cavity of a live mouse [102]. An innovative technique for the precise control of a low amount of fluorescent-dye-coated magnetic nanoparticles that formed a swarm that effectively amplified the concentration of the dye within a specific region of a cell was presented (Figure 8f) [103]. This technique enabled the swarm produced inside the cell to exhibit a signal-to-noise ratio that was 10-fold greater than the traditional global dye treatment method, offering a powerful tool for intracellular treatment. Photoacoustic imaging (PAI) has gained popularity in the past decade because of the distinct discrimination between the structures and ample choices of contrast agents [24]. PAI was reported to include the use of the 3D real-time detection and tracking of cell-sized nickel-based magnetic microrobots in the mouse brain vasculature [107]. A novel light-triggered assembly of gold nanoparticles was reported to enhance the contrast and the image quality for the PAI of tumors in vivo [108]. A polydopamine-coated magnetized *Spirulina*-based microswimmer was fabricated to enhance the photoacoustic (PA) signal, making the PA real-time image trackable [67].

## 5. Conclusions and Outlook

In this review, different kinds of biodegradable microrobots, their degradation mechanisms, and their typical applications are summarized.

Because of the biosafety of these microrobots, they have great potential in several biomedical applications, for example, targeted delivery toward different kinds of tumor microenvironments, delivering drugs to the eye, knee cartilage regeneration [70], enhanced chemodynamic therapy [109], spinal cord stimulation [110], sensing [111], treating acute ischemic stroke [112], and hyperthermia therapy [113,114]. Biodegradable microrobots are one of the most critical development trends in this area. Further in-depth studies on the biodegradability of microrobots are needed.

There are different unsolved challenges in the clinical application of microrobots [115,116,117,118]. The first challenge involves manufacturing [119,120]. Conventional fabrication methods are usually time-consuming, expensive, and difficult to mass produce, and they are not practical in clinical practice. The second challenge involves control. Biodegradable microrobots need to be controlled precisely to perform their intended functions. To operate microrobots, a common method is collaborating with an imaging system to navigate the microrobot. Automated control systems need to be developed nowadays. Precisely tuning the degradation rate is also a challenge. Whether microrobots can be effectively degraded in the human body and whether the metabolites will not cause additional impacts on the human body still need further experimental verification. Most of the microrobots in existing experiments are completely degraded after a certain period of time under a certain concentration of enzyme solution or PBS solution. However, the composition of the human environment is more complex; these concentrations do not necessarily correspond to the actual situation in the human body, and the exact time for complete degradation is unpredictable. The degradation rate of biodegradable microrobots shall be carefully controlled to ensure that they are effective during their intended lifespan. Meanwhile, some metal-based microrobots are degraded into cations, such as Zn^2+^, Ga^3+^, and Mg^2+^. Their intake beyond a certain range may cause harm to the human body. Moreover, in order to realize the successful degradation of microrobots, specific requirements have to be satisfied. For instance, acid-degraded microrobots are mainly limited to treating stomach diseases. Fourth, a single microrobot can only carry a limited amount of drugs or cells. Furthermore, it is challenging to design microrobots that can address the non-Newtonian rheological behaviors of biological fluids [121,122,123,124,125].

To tackle these challenges, developing novel fabrication methods, such as two-photon direct writing, has been applied in recent years and could be a breakthrough in the field. Diverse in vivo experiments to examine the appropriate lasting time of drug release and microrobot degradation are needed, and a longer experimental period is needed to test the toxicity of the metabolites to animals or humans. To develop automated control systems, there is considerable enthusiasm for integrating machine learning techniques into the control of biodegradable microrobots, allowing them to execute sophisticated tasks without explicit programming [126,127,128,129,130,131]. It is promising to develop more degradation mechanisms that have a wider scope of applications, and developing biodegradable microswarms is a good choice to address this problem [132,133]. The potential benefits of biodegradable microrobots in healthcare make these challenges worth addressing.

## Data Availability

Not applicable.
